# Isolation, identification, and selection of strains as candidate probiotics and starters for fermentation of Swedish legumes

**DOI:** 10.29219/fnr.v64.4410

**Published:** 2020-09-02

**Authors:** Inger-Cecilia Mayer Labba, Thomas Andlid, Åsa Lindgren, Ann-Sofie Sandberg, Fei Sjöberg

**Affiliations:** 1 Division of Food and Nutrition Science, Department of Biology and Biological Engineering, Chalmers University of Technology, Gothenburg, Sweden; 2 Department of Clinical Bacteriology, Institute of Biomedicine, Sahlgrenska Academy, University of Gothenburg, Gothenburg, Sweden; 3 Department of Infectious Diseases, Institute of Biomedicine, Sahlgrenska Academy, University of Gothenburg, Gothenburg, Sweden

**Keywords:** isolation, fermentation, fava bean, pediococcus pentosaceus, phytate

## Abstract

**Background:**

The non-dairy sector is growing, fermented alternatives to dairy are sparse. Adapted starter cultures to substituting raw materials needs to be developed.

**Objective:**

Aims of this study were to isolate, identify, and phenotypically characterize lactic acid bacteria (LAB) that inhabit Swedish legumes, and assess properties necessary for selecting strains with the ability to ferment a bean beverage and with potential health beneficial properties.

**Design:**

Isolates of presumed LAB were obtained from legumes collected at Öland, Sweden. Strain diversity was assessed by repetitive polymerase chain reaction (rep-PCR). The strains were identified using matrix-assisted laser desorption/ionization–time-of-flight mass spectrometry (MALDI-TOF MS). Species belonging to *Enterococcus* were predominant along with *Pediococcus* and closely related *Bacillus*. Strains were tested for tolerance to low pH, phenol, and bile as well as their bile salt hydrolase (BSH) activity. In addition, *Enterococcus* strains were tested for antibiotic resistance, and *Pediococcus* strains for their ability to ferment a bean beverage.

**Results:**

From the 25 strains characterized, five were found resistant to low pH, bile, and phenol, suggesting that they can survive a passage through the gastrointestinal tract (GIT) and hence potentially exert beneficial effects in the host. These are suggested for further investigation on specific host-beneficial properties. Two of these, belonging to *Pediococcus pentosaceus,* were able to ferment a bean beverage without any added nutrients, indicating that the *Pediococcus* strains are well adapted to the bean substrate. One of the P. *pentosaceus* strains were also able to markedly improve the reduction of phytate by the phytase-producing yeast strain *Pichia kudriavzevii* TY1322 during co-fermentation as well as increase the final cell count of the yeast strain.

**Conclusion:**

Strain isolation and characterization performed in this study aids in selecting starter cultures for legume fermentation. Nutritional properties can be improved by co-fermentation with yeast indicating that novel nutritious fermented non-dairy products could be developed.

## Popular scientific summary

- Demand for fermented non-dairy products is increasing, there is a need to develop adapted starter cultures.
- We isolated 25 lactic acid bacteria strains from legumes. The strains were characterized for potential probiotic properties and tested for their ability to ferment a bean beverage.
- *Pediococcus* strains could grow in bean beverage and improve bioavailability of minerals in co-fermentation with yeast.
- Results indicate that new and improved fermented non-dairy products could be developed.

Pulses, such as beans and lentils, have a favorable nutritional composition with high contents of protein, dietary fibers, minerals, vitamins, and phytochemicals, and positive health effects have been demonstrated (1). Furthermore, pulses have been suggested as alternatives to resource-demanding animal products, and could contribute to climate change mitigation by lowering greenhouse gas emissions in arable systems (2).

Foods with a low environmental impact need to become more accessible, economically sustainable, and attractive for both farmer and consumer. In the Scandinavian countries, where cultivation of soy is not suited to the climate, other domestic protein crops, such as fava beans, have a large potential in partly replacing animal products. This raises the need to develop new palatable food products with low environmental impact. Fermentation is a well-known process with low energy use that can provide several positive nutritional and sensory properties to raw materials such as grain legumes (3, 4). Depending on the organisms used, the end product will have different properties and nutritional values (4). Previous studies have shown that certain microorganisms can synthesize vitamins such as folic acid, riboflavin, niacin, thiamin (5, 6), and B12 (7) using grain legumes as a substrate. Some bacteria are able to metabolize at least two of the main components (n-hexanal and pentanal) that give a bean-like flavor to soy milk (8). Furthermore, fermentation lowers the level of oligosaccharides, which can cause postprandial flatulence from legumes (9, 10), and other anti-nutritional components such as tannins, phytate (11) , and trypsin inhibitors (12). Different strains within the same species can have completely distinct metabolic patterns, which, in turn, affect not only synthesis of vitamin and breakdown of undesirable components but also production of substances that affect taste and texture of the product, such as exopolysaccharides (13).

Lactic acid bacteria, LAB, are a vast group of bacteria within the phylum Firmicutes and the class Bacilli, showing considerable genotypic and phenotypic diversity. The closely related genus *Bacillus* belongs to the same class (Bacilli) and hence related to LAB. Both LAB and *Bacillus* include probiotic as well as pathogenic organisms. LAB are the most used bacteria within the food sector and many strains have been graded as Generally Recognized as Safe (GRAS) strains. Despite generally not being able to reduce the levels of phytate, LAB can provide favorable conditions for phytase activity and contribute indirectly to an improved phytate reduction important to improve bioavailability of essential dietary minerals, such as iron and zinc, in cereals and legumes (14). Co-fermentation between LAB and phytase-producing yeast strains takes place in many natural food fermentations (15, 16). Co-fermentation between LAB and yeast has been suggested to improve sensory properties and prolong shelf life as well as improve resistance to spoilage molds (17). This indicates interesting applications within the food industry for new strains of LAB.

Despite already extensively used in the food industry, there is still a need to find and develop starter cultures functionally adapted to different types of plant-based raw material as alternative to dairy products. Apart from the functional and nutritional improvements during fermentation, LAB can also have probiotic properties. According to the World Health Organization (WHO), probiotics are ‘live microorganisms which, when administrated in adequate amounts, confer a health benefit to the host’ (18). Health benefits include reduction in hypercholesterolemia (19), reduction in symptoms of irritable bowel syndrome (20, 21), normalization of disturbed gut microbiota, regulation of intestinal transit, competitive exclusion of pathogens, and production of short chain fatty acids (22). The importance of gut microbiota in health and disease has become increasingly evident, and is reflected in the popularity of fermented and probiotic foods among consumers and their rapid market growth (23). The gut microbiota can be modified by the food consumed, for instance by dietary fiber present in legumes, as well as live microbes present in fermented food (24). New probiotics should be selected based on their strain characteristics. The main selection criteria for probiotics include many functional properties, such as tolerance to gastric acidity, bile, and phenol toxicity and ability to de-conjugate bile salts (25). In the present study, we searched for, and initiated studies of, strains that efficiently metabolize complex carbohydrates in pulses, with the long-term goal to develop new types of digestible, palatable, and nutritious fermented legume-based foods.

The aim was to identify and isolate strains with the ability to ferment a fava bean beverage and to characterize LAB isolated from Swedish grain legumes, perform co-fermentation to reduce phytate, a major inhibitor of iron and zinc absorption, and initial test of probiotic potential of the strains.

## Materials and methods

### Plant samples

A total of 23 samples were collected from fields (*n* = 12) and from stored seeds (*n* = 11) derived from plants cultivated during 2015. The 12 field samples came from five different locations of Öland, Sweden. On each site, approximately 50 g were collected by picking whole pods directly from the plant with sterile gloves. Samples were placed in clean bags and stored at 4°C until they were used for analysis. The remaining 11 samples were seeds that were harvested at Öland, dried and stored at 4°C for 8 months by the producer. The samples were different varieties of *Phaseolus vulgaris* (black beans, brown beans, kidney beans, navy beans, borlotti beans, and yin yang), and two varieties of *Pisum sativum* (grey and yellow peas).

### Isolation of LAB

For the selection of LAB from the bean samples, about 1 g of bean pods were aseptically transferred to de Man, Rogosa and Sharpe (MRS) broth (Sigma-Aldrich, Missouri, USA) with pH adjusted to 5.7 using HCl (Sigma-Aldrich). The flasks were incubated at 37°C for 4 days in a shaking incubator (Barnstead Lab-Line, ON, Canada) at 190 rpm. Dilutions of the liquid were spread in parallels on MRS agar plates (Scharlab, Spain); these were then incubated at anaerobic conditions using Anaerocult^®^ A (Merck Millipore, Germany) at 37°C for 72 h. Colonies were picked out and re-streaked on MRS agar plates until isolated bacterial strains were obtained. Strain isolation was made for two subsequent times for each sample.

### Identification of isolates

Colonies that were Gram-positive, oxidase-negative, and catalase-negative were selected for further analyses. These isolates were harvested for DNA extraction and stored at –80°C.

#### Repetitive polymerase chain reaction (rep-PCR) genomic fingerprinting

To minimize the risk of duplicate strains, we performed rep-PCR. Genomic DNA was extracted from 116 isolates according to a previously described protocol (26). One loop of bacteria incubated at 39°C for 74 h on horse blood agar plates (Scharlab) were inoculated in 50-µL lysis buffer (10 mmol/L Tris HCl, 1 mmol/L EDTA, and 10 mmol/L saline; Sigma-Aldrich) and incubated for 10 min at 95°C. Samples were then centrifuged for 5 min at 12,000 × g. The supernatant was collected, measured for DNA concentration using a NanoDrop 2000c spectrophotometer (Thermo Fisher Scientific, Massachusetts, USA), and stored at −20°C until used.

Strain diversity was assessed by rep-PCR using the single nucleotide (GTG)_5_ primer (5’-GTGGTGGTGGTGGTG-3’) (Invitrogen, California, USA) (27). Reactions were performed using 2,720 thermal cycler (Applied Biosystems, California, USA). The amplification program had an initial denaturation of 98°C for 3 min, followed by 30 cycles of denaturation (98°C, 30 s), annealing (40°C, 1 min), extension (65°C, 8 min), and a final single extension (65°C, 16 min). Each 25 µL of PCR reaction contained 1x HF Phusion PCR buffer (Thermo Fisher), 2.5 mmol MgCl_2_ (Thermo Fisher), 170 µg/mL BSA (Thermo Fisher), 200 µmol/L dNTPs (Thermo Fisher), 2 µmol/L (GTG)_5_ primer (Invitrogen), 0.05 U Phusion polymerase (Thermo Fisher), 5% Dimethyl sulfoxide (DMSO; Thermo Fisher), and 60 ng of template DNA.

Amplification products were resolved by electrophoresis on a 25-cm gel in a 40-cm cell (Bio-Rad, Sub Cell GT, California, USA) at a constant voltage of 1.5 V/cm for 16 h in 4-mm 1.5% (w/v) agarose (Thermo Fisher) gel in 1x TBE buffer (Sigma-Aldrich). A DNA molecular weight ladder (Thermo Fisher) was used as a reference. Gels were stained post-run with 3x Gelred in 0.1 M NaCl (Biotium, California, USA) and visualized by UV light using a Gel Doc (BioRad).

The PCR fingerprints were analyzed using BioNumerics (Applied Maths NV, Sint-Martens-Latem, Belgium). Similarity of digitalized band patterns was calculated using the Sørensen–Dice coefficient. Complete linkage algorithms were used to construct an average linkage dendrogram to show relationship of isolates.

#### MALDI-TOF MS identification of isolates

The 116 bacterial isolates were analyzed twice with matrix-assisted laser desorption ionization–time-of-flight mass spectrometry (MALDI-TOF MS) analysis using intact cell biomass obtained from overnight cultures of LAB at 37°C on horse blood agar plates. Samples were spotted in duplicates on disposable target plates (bioMeriéux, Marcy l’Etoile, France) and overlaid with 1 µL VITEK^®^ MS-FA (Formic acid; bioMeriéux), then air-dried and again overlaid with 1 µL VITEK^®^ MS-CHCA matrix solution (α-cyano-4-hydroxycinnamic acid; bioMeriéux). MALDI-TOF MS analysis was performed using a VITEK^®^ MS instrument (bioMeriéux) in the mass range of 2,000–20,000 m/z, and the spectral data were analyzed using the VITEK^®^ MS IVD and VITEK^®^ MS SARAMIS/RUO databases (bioMeriéux).

### Phenotypic tests related to probiotic potential

A number of prerequisites need to be fulfilled for a strain to be considered a candidate for further investigation of its probiotic properties. We studied the ability of strains to survive the harsh conditions such as in the gastro-intestinal tract (GIT), including tolerance to bile, low pH, and phenol.

#### Tolerance to low pH

Survival of strains in acidic conditions was asessed by incubation in low pH growth media according to a previously described method (28) using pH 2.5. Unique strains, which we identified by MALDI-TOF, were propagated twice in MRS broth for 24 h at 37°C, collected by centrifugation (at 5,000 × g, for 5 min), and washed twice in phosphate buffer solution (PBS; Thermo Fisher). After propagation and dilution, cells were inoculated in triplicates into 8 mL of MRS broth adjusted to pH 2.5 to simulate gastric conditions. Cells were then incubated at 37°C for 2.5 h with continuous stirring using a tube rotator (New Brunswick Scientific, New Jersey, USA). Bacterial enumeration was performed using five replicates per dilution on MRS agar plates at 0, 0.5, 1, 1.5, 2, and 2.5 h.

#### Phenol tolerance

The isolates’ ability to survive in the presence of phenol, which can be present in the gut, was examined according to a previously described method (29), with minor modifications. Unique strains were prepared in the same way as described for the low pH test. The OD_600_ was adjusted to 0.4–0.6, then 200 μL of the diluted cells were inoculated in duplicates into 8 mL of MRS broth, pH 5.7, with 0.4% phenol (Sigma Aldrich). The cells were then incubated in a shaking incubator at 37°C for 24 h. Bacterial enumeration was performed using five replicates per dilution on MRS agar plates at 0 and 24 h.

#### Bile tolerance

Tolerance of the isolates to bile salts was assayed as described by Gilliland et al. with slight modifications (30). Prior to the bile tolerance test, strains were prepared in the same way as described for the low pH test. The OD_600_ was then adjusted to 0.4–0.6 with PBS, and 200 μL of the diluted cells were inoculated in duplicates into 8 mL of MRS broth with 0.3% (w/v) bile salts (Sigma Aldrich). Cells were then incubated in a shaking incubator at 37°C for 48 h. The bacterial suspension was assessed as colony forming units/milliliter (CFU/mL) using five replicates on MRS agar plates at 0, 24, and 48 h.

#### Qualitative determination of bile salt hydrolase (BSH) activity

The taurodeoxycholic acid (TDCA) hydrolase activity was determined for unique strains using 0.5% (w/v) bile salt–MRS agar plate assay (31). MRS agar plates were supplemented with 0.5% TDCA (Sigma Aldrich).

Bile salt–MRS agar plates were plated in duplicates with each representative strain and incubated anaerobically using an Anaerocult^®^ A at 37°C for 72 h before observation. A precipitation zone surrounding colonies indicated BSH activity.

#### Screening of isolated *Enterococcus* strains for antibiotic resistance

Isolates identified as *Enterococcus* species were tested for antibiotic resistance using the disk diffusion test according to the Clinical and Laboratory Standards Institute (CLSI) (32). The isolated *Enterococcus* was tested for resistance to ampicillin, trimethoprim, vancomycin, and nitrofurantoin.

#### Production of bean flour

Dried fava beans were soaked overnight in deionized water with 2% (w/v) NaHCO_3_ (Sigma Aldrich), then peeled by hand and incubated at 95°C in deionized water with 0.5% (w/v) NaHCO_3_ for 4 min in a water bath. The boiled beans were then blended in 90°C deionized water for 60 s and put on an ice bath to cool before freeze-drying. The freeze-dried flour was then stored at −18°C until use.

#### Fermentation of fava bean flour medium by *Pediococcus pentosaceus*

A bean flour medium was prepared using deionized water with 6% (w/v) fava bean flour brought to a boil, with pH adjusted to 6.2, filtered through a cheese cloth and autoclaved. Selected strains belonging to *Pediococcus pentosaceus* were pre-cultured overnight in liquid MRS at 37°C in a shaking incubator. The cells were washed twice in PBS and diluted in PBS to an OD_600_ of 0.5–0.6. In triplicates, 8 mL of the fava bean medium were inoculated with 200 μL of diluted cells from five selected strains. Bacterial enumeration was performed using five replicates per dilution on MRS agar plates at 0, 4, 6, 8, 12, 24, and 48 h.

#### Co-fermentation of fava bean flour medium

Bean flour medium was prepared as described for fermentation by *P. pentosaceus. Pichia kudriavzevii* TY1322 (33) *and P. pentosaceus* strain 77 were pre-cultured overnight in liquid MRS and yeast extract peptone dextrose (YPD) medium respectively at 37°C and 220 rev/min in a rotary shaker. Cells were washed twice in PBS and diluted to an OD_600_ of 0.55–0.60. In triplicates, 80 mL of the fava bean medium were inoculated in three combinations: (1) 600 μL of diluted yeast cells + 600 μL of diluted bacterial cells, (2) 600 μL of diluted bacterial cells, and (3) 600 μL of diluted yeast cells. Microbial enumeration was performed using five replicates per dilution on agar plates at 0, 4, 6, 8, 12, 24, 36, 48 h. YPD agar plates with 100-mg/L chloramphenicol (Sigma-Aldrich) were used for combinations 1 and 3. MRS agar plates with 0.5-mg/mL cycloheximide (Sigma-Aldrich) were used for combination 1 and 2. Each time, set microbial enumeration was performed and samples for phytic acid analysis were withdrawn from the fermentation media.

#### Phytate analysis

Fava bean flour medium (500 μL) was collected during fermentation and immediately mixed with 500 μL of 1 M HCl to stop enzymatic and microbial activity. Samples were stored at −20°C until analysis. Samples were thawed and mixed using a laboratory shaker (Heidolph Reax 2; Heidolph Instruments GmbH, Schwabach, Germany) for 3 h, centrifuged, and the supernatant was transferred to an HPLC vial. Phytate was analyzed as inositol hexaphosphate (InsP_6_) by high-performance ion chromatography (HPIC) according to Carlsson et al. (34). The chromatography setup consisted of an HPLC pump (model PU-4080i; Jasco Inc., Easton, MD, USA) for the eluent and an RHPLC pump (model PU-4180; Jasco) equipped with a PA-100 guard column and a CarboPac PA-100 column.

InsP_6_ was eluted with an isocratic eluent of 80% HCl (1 M) and 20% H_2_O at 0.8 mL/min, subjected to a post-column reaction with ferrous nitrate, and detected with at 290 nm in a UV-visible HPLC detector (UV-4075; Jasco). Each sample had a run time of 7 min, and the InsP_6_ concentration was calculated using external standards covering the concentration range of 0.1–0.6 μM/mL.

## Results

### Isolation and identification by Rep-PCR and MALDI-TOF MS

A total of 116 isolates of putative LAB were originally obtained from 23 legume samples. Strains fulfilling all criteria used to select LAB and closely related lactate-producing bacteria were isolated from each legume, except from the samples of black beans, from which we could not isolate any LAB. Using rep-PCR fingerprints, the 116 isolates were clustered into five main groups. Of these, fingerprints showing >90% similarity were assigned to the same genotype and were considered a cluster, as suggested by Maluping et al. (35).

**Fig. 1:**
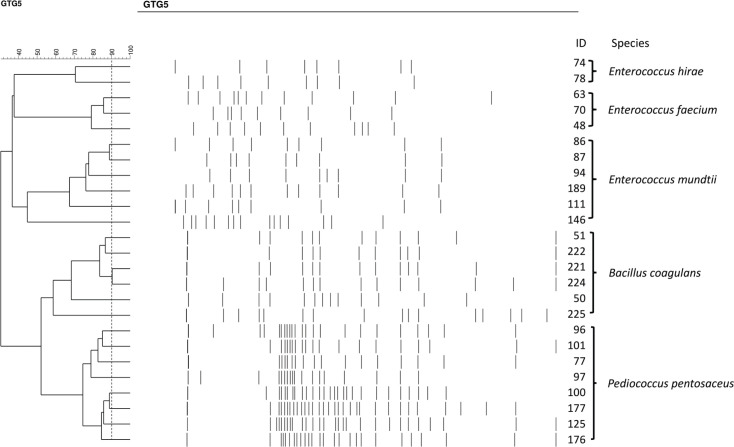
Dendrogram generated after cluster analysis of the digitalized (GTG)_5_-PCR fingerprints of the isolated strains. Cluster analysis was made using the Sørensen–Dice coefficient and a complete linkage algorithm.

From each cluster, one strain was randomly picked as a representative of the group for further analysis, resulting in a total of 25 unique strains (Fig. 1). MALDI-TOF MS identified the strains as *Enterococcus hirae*, *Enterococcus faecium, Enterococcus mundtii, Bacillus coagulans*, and *P. pentosaceus.*

The collected borlotti beans were found to have the highest LAB diversity, with strains from almost all identified genera present. Conversely, no LAB was isolated from black beans despite repeated enrichments. In total, eight unique strains of *P. pentosaceus* and six unique strains of *B. coagulans* were found from the plant samples (Table 1).

**Table 1:** Isolated strains of LAB and *Bacillus* and their origin

Species	Isolated from	Number of unique strains
*Bacillus coagulans*	KB**, YY*	6
*Enterococcus hirae*	BoB‡, GP§	2
*Enterococcus faecium*	BoB, YP¶, KB, WB†	3
*Enterococcus mundtii*	BoB, BB†	6
*Pediococcus pentosaceus*	YY, BB, BoB	8
Total number of strains	25	-

* YY = yin yang beans;

† BB = brown beans;

‡ BoB= borlotti beans;

§ GP = grey peas;

¶ YP = yellow peas

** KB= kidney beans;

†† WB = white beans.

### Phenotypic tests related to probiotic potential

#### Tolerance to low pH

**Fig. 2:**
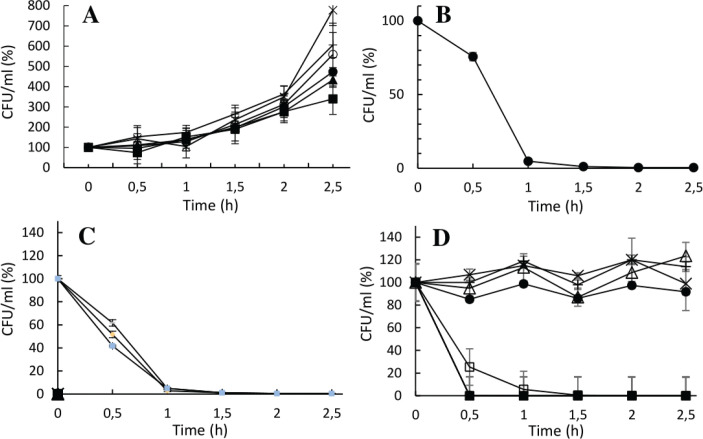
Cell concentration as a function of incubation time during low pH stress (pH 2.5) in MRS. Data presented as percentage of the initial CFU/mL (set as 100%). Graph A: *B. coagulans*; graph B: *E. faecium*; graph C: *E. mundtii*; and graph D: *P. pentosaceus.* Symbols correspond to different strain numbers. Graph A, *B. coagulans*: 50 (●), 51 (▲), 221 (■), 222 (×), 224 (+), 225 (○)*;* graph B, *E. faecium*: 63 (●); graph C, *E. mundtii*: 94 (●), 146 (▲), 189 (■); and graph D, *P. pentosaceus*: 77 (●), 96 (▲), 97 (■), 100 (×), 101 (+), 125 (○), 176 (∆), 177 (□).

Each of the unique *B. coagulans* strains showed a high degree of tolerance to pH 2.5 and had an exponential growth during the entire incubation (Fig. 2A). Strains belonging to *E. faecium* and *E. mundtii* showed a generally low tolerance to pH 2.5 and were completely depleted after incubation (Figs 2B and 2C). *P. pentosaceus*strains (Fig. 2D) showed two distinctive growth patterns; strain number 77, 100, 101, and 176 showed tolerance to low pH with small variations of CFU/mL. On the contrary, *P. pentosaceus* strains 96, 97, and 177 did not survive incubation (Fig. 2D).

#### Phenol tolerance

**Fig. 3:**
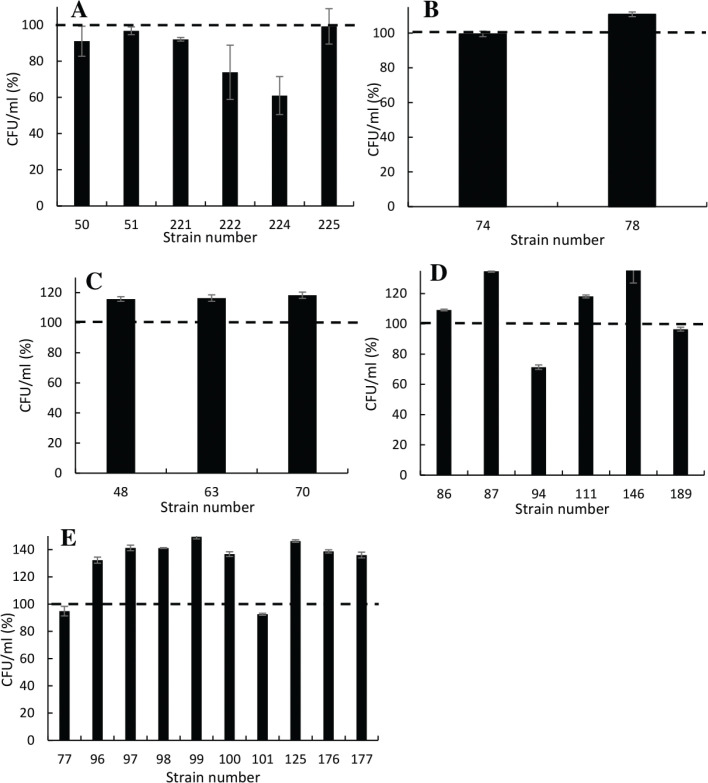
Survival of the isolated 25 unique strains inoculated in liquid MRS supplemented with 0.4% phenol. Bars show the percentage of CFU/mL after 24 h of incubation; dash line indicates the starting CFU (normalized to 100%). Graph A, *B. coagulans*; graph B, *E. hirae*; graph C, *E. faecium*; graph D, *E. mundtii*; and graph E, *P. pentosaceus*.

Overall, strains from all genera showed tolerance to 0.4% phenol with a few exceptions. These were *B. coagulans* strains 222 and 224 (Fig. 3A) and *E. mundtii* strain 94 (Fig. 3D). In this experiment, strains 222 and 224 showed a decrease of 30–40% in CFU/mL. Among other isolates, *E. mundtii* strains 87, 111, and 146 showed an average increase of 29% in CFU/mL (Fig. 3D). Strains belonging to *P. pentosaceus* (Fig. 3E) exhibited a high tolerance with a consistent level of CFU/mL before and after incubation for strains 77 and 101, while strains 96–100, 125, 176, and 177 showed an average increase of 39% in CFU/mL.

#### Bile tolerance

**Fig. 4:**
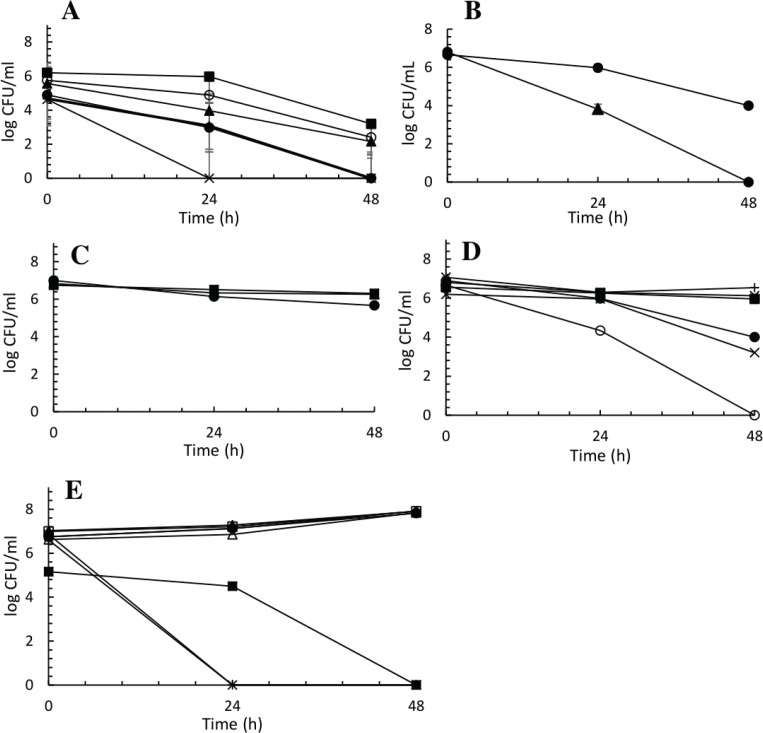
Survival curves of 25 unique strains identified by MALDI-TOF, inoculated in liquid MRS supplemented with 0.3% bile. Graph A: *B. coagulans*; graph B: *E. hirae*; graph C: *E. faecium*; graph D: *E. mundtii*; and graph E: *P. pentosaceus.* Symbols correspond to different strain numbers. Graph A, *B. coagulans*: 50 (●), 51 (▲), 221 (■), 222 (×), 224 (+), 225 (○); graph B, *E. hirae*: 74 (●), 78 (▲); graph C, *E. faecium*: 48 (●), 63 (▲), 70 (■); graph D, *E. mundtii*: 86 (●), 87 (▲), 94 (■), 111 (×), 146 (+); and graph E, *P. pentosaceus*: 77 (●), 96 (▲), 97 (■), 100 (×), 101 (+), 125 (○), 176 (∆), 177 (□).

*B. coagulans* (Fig. 4A) and *E. hirae* (Fig. 4B) strains showed a lowered viability after 24 h of incubation, followed by a larger drop in viability at 48 h of incubation with half of the strains completely depleted. Strains belonging to *E. faecium* showed a consistently high tolerance to 0.3% bile acid after 48 h of incubation, with an average decrease of 11% in log CFU/mL (Fig. 4C). In the experiment, *E. mundtii* strains had inter-specific differences in response to bile acid. Strains 87, 94, and 146 (Fig. 4D) showed a high level of tolerance with a viable cell count close to the initial concentration; on the contrary, *E. mundtii* strains 86, 111, and 189 showed a low level of tolerance, where cells belonging to strain 189 was completely depleted after 48 h of incubation. Similar to the *E. mundtii* strains, *P. pentosaceus* (Fig. 4E) strains 77, 96, 125, 177, and 176 had a small average increase, while strains 97, 99, 100, and 101 were completely depleted after incubation.

#### Qualitative determination of BSH activity

None of the strains belonging to *B. coagulans* and *P. pentosaceus* showed BSH activity, while all strains from *E. faecium* and *E. mundtii* showed BSH activity. Among *E. hirae,* we found both BSH-negative and BSH-positive strains (Table 2).

**Table 2:** Bile salt hydrolase (BSH) activity assessed in all selected species

Species	BSH activity
*Bacillus coagulans*	-
*Enterococcus hirae* 74	-
*Enterococcus hirae* 78	+
*Enterococcus faecium*	+
*Enterococcus mundtii*	+
*Pediococcus pentosaceus*	-

Differences within a species were only found in *E. hirae.*

#### Antibiotic resistance

From the 11 screened *Enterococcus* strains, four strains (strains 63, 94, 146, and 189) showed susceptibility to all tested antibiotics. Of the remaining strains, three showed resistance toward one or more of the tested antibiotics and four showed intermediate resistance to at least one of the tested antibiotics (Table 3).

**Table 3:** Resistance toward tested antibiotics measured as antibiotic disk diffusion zones (in millimeters)

Isolate	Ampicillin	Trimethoprim	Vancomycin	Nitrofurantoin
*E. hirae* 74	4 (R)	6 (R)	29 (S)	42 (S)
*E. hirae* 78	19 (S)	31 (S)	16 (I)	16 (I)
*E. faecium* 48	19 (S)	3 (R)	16 (1)	13 (R)
*E. faecium* 63	19 (S)	27 (S)	17 (S)	11 (S)
*E. faecium* 70	19 (S)	27 (S)	17 (S)	16 (I)
*E. mundtii* 86	18 (S)	3 (R)	16 (S)	15 (I)
*E. mundtii* 87	19 (S)	31 (S)	16 (I)	16 (I)
*E. mundtii* 94	21 (S)	41 (S)	19 (S)	21 (S)
*E. mundtii* 111	19 (S)	34 (S)	16 (I)	17 (S)
*E. mundtii* 146	23 (S)	32 (S)	17 (S)	19 (S)
*E. mundtii* 189	22 (S)	44 (S)	19 (S)	25 (S)

Clinical and Laboratory Standards Institute (CLSI) interpretive categories are given in parentheses: S, susceptible; I, intermediate; and R, resistant.

#### Fermentation of fava bean flour medium by *P. pentosaceus*

**Fig. 5:**
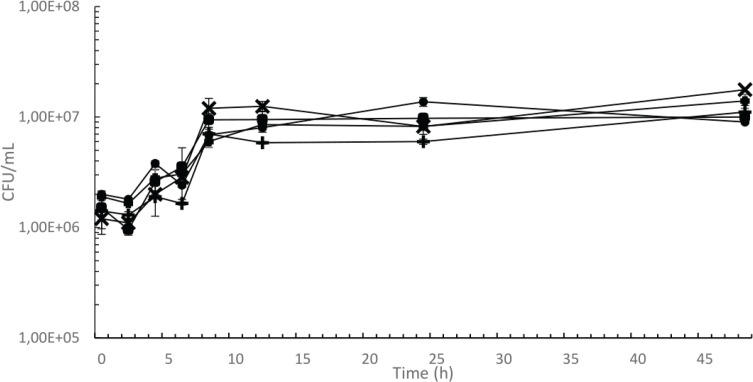
Growth curve of selected *P. pentosaceus* strains in fava bean beverage without additional carbon sources. Strain numbers and their symbols are: 77 (●), 96 (▲), 97 (■), 100 (×), 176 (+). Error bars represent standard errors.

Each of the isolated and unique *P. pentosaceus* strains was able to grow in 6% fava bean medium without addition of carbon or any other nutrients. The strains showed a growth pattern with an initial lag phase followed by exponential growth. During the exponential phase, the strains differed in generation time. Strains 97 and 100 had a generation time of approximately 1 h, calculated by regression of data points between 2 and 8 h. Strains 77 and 96 had a generation time of approximately 2 h during the same time frame. Strain 176 had an intermediate generation time of 1.4 h under the given conditions. All strains reached a stationary phase after 8 h of incubation (Fig. 5).

## Co-fermentation of fava bean flour medium by P. pentosaceus 77 and P. kudriavzevii TY1322

**Fig. 6:**
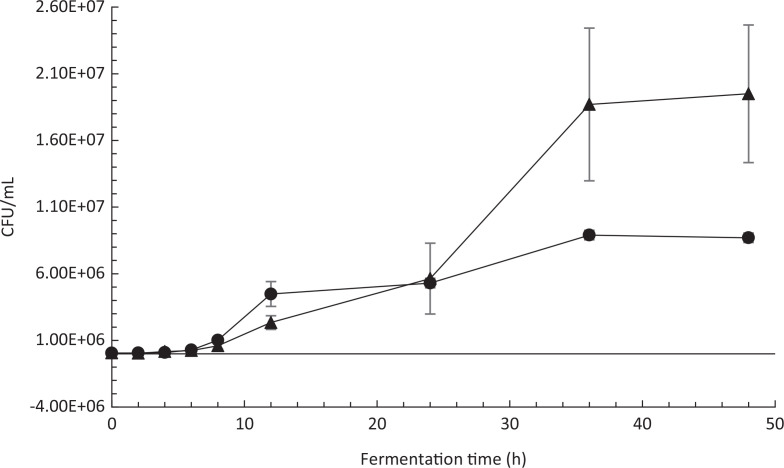
Growth curve of *P. kudriavzevii* TY1322 in monoculture and in co-fermentation with *P. pentosaceus* strain 77 in fava bean medium. Symbols correspond to: *P. kudriavzevii* TY1322 in monoculture (●) and in co-fermentation (▲). Error bars represent standard errors.

**Fig. 7:**
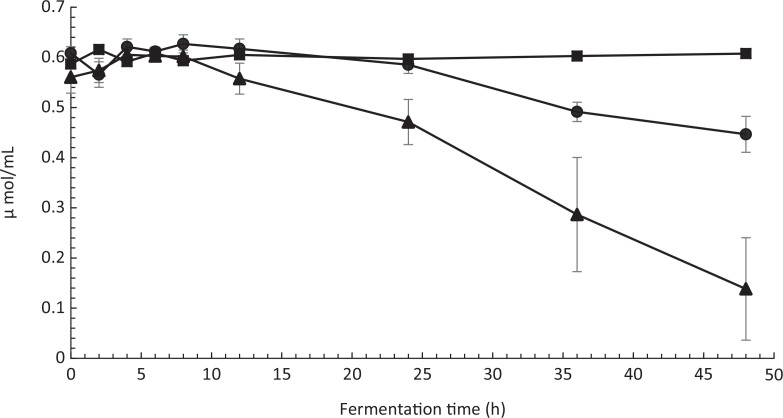
Phytate content in fava bean medium fermented by *P. kudriavzevii* TY1322 (●), *P. pentosaceus* strain 77 (■), and by co-fermentation of both *P. kudriavzevii* and *P. pentosaceus* (▲)*.* Error bars represent standard errors.

Yeast strain *P. kudriavzeviiI* TY1322 showed improved growth during co-fermentation with *P. pentosaceus* strain 77 compared to fermentation in monoculture. In both monoculture and co-fermentation, the yeast strain reached a stationary phase after 36 h, but in co-fermentation, the cell count of *P. kudriavzeviiI* TY1322 was 124% higher compared to fermentation in monoculture (Fig. 6). The reduction of phytate in the fava bean medium by *P. kudriavzevii* TY1322 was significantly improved using a co-starter culture compared to fermentation monoculture (Fig. 7). After 48 h of fermentation, the phytate content in the medium fermented by *P. kudriavzevii* TY1322 in monoculture had decreased by 25%. The phytate content in the medium fermented by a mixture of *P. kudriavzevii* TY1322 and *P. pentosaceus* 77 was reduced by 77%. *P. pentosaceus* strain 77 was not able to reduce the phytate content by monoculture.

## Discussion

There are strong arguments to change the food patterns in industrialized countries into more healthy and sustainable ones. Pulses, such as beans and lentils, are often described as a nutritious and sustainable protein source that could be used as alternatives to meat and dairy products. Fermentation of legumes improve digestibility, natural preservation, and raise their nutritional value. Certain microbial strains may also be probiotic.

Today, despite the growing demand for dairy-free products, only very few fermented non-dairy products exist in the market worldwide, and few strains available commercially are aimed for the fermentation of plant-based foods (36).

In the present study, 116 isolates were obtained. Subsequently, they were sorted into 25 unique strains from three different genera as confirmed by rep-PCR and MALDI-TOF MS. The strains were identified as LAB, and closely related *B. coagulans*. Since *B. coagulans* is a spore forming bacteria, it can survive extreme conditions and has previously been suggested as an health-promoting ingredient (37). Owing to the spore forming ability, *B. coagulans* can survive human ingestion and reach the intestine where it germinates. In the intestine, it can contribute to positive effects such as production of short-chain fatty acids, promoting populations of beneficial bacteria (38), and aid in the digestion of protein and carbohydrates (39). The ability to increase protein bioavailability is of relevance in the ongoing protein shift toward more plant-based protein. Probiotic food products containing *B. coagulans* are currently sold to consumers commercially (37), and *B. coagulans* has received the Generally Recognized as Safe (GRAS) status and is reported as safe by the US Food and Drug Administration (FDA) (40).

The 25 unique strains were characterized *in vitro* in order to select candidate strains suitable for further investigation related to probiotic properties. Strains belonging to *E. faecium, E. mundtii*, and *P. pentosaceus* showed a high level of tolerance to 0.3% bile. Tolerance to bile and low pH is important to evaluate, since it indicates survival during the passage through the upper GIT, a necessary step for probiotic organisms delivered via food (41). Among the strains isolated in this study, strains 77, 100, 101, and 176 belonging to *B. coagulans* and *P. pentosaceus* have shown tolerance to pH 2.5.

Tolerance to phenol was evaluated, since phenols may be formed in the gut by bacterial deamination of aromatic amino acids derived from dietary or endogenously produced proteins. Phenols and other products of protein degradation in the colon, such as ammonia, indoles, and amines, exert toxic effects in animal models and *in vitro*. These compounds are present in fecal samples and can exert bacteriostatic effects against some LAB (42).

We examined antibiotic resistance for *Enterococcus* strains because of the high occurrence of resistance within the *Enterococcus* genus, and because of the risk of pathogenicity. In the future studies, the presence of virulence genes should be addressed. Four of the *Enterococcus* strains (*E. faecium* 63 and *E. mundtii* 94, 146, and 189) were susceptible to all four tested antibiotics. Owing to their tolerance to salts and acids, *Enterococcus* strains are highly adapted to surviving in the GIT. Some *Enterococcus* strains have interesting properties relevant from food technology and medical perspective, such as the ability to produce multiple bacteriocins (43). Even though a few *Enterococcus* strains are currently commercialized as probiotics and used for treatment and prevention of certain diseases in humans and animals, these strains might originate from bovine feces used as manure in the fields, and may not be suitable to metabolize legumes (43, 44).

The ability to produce BSH enzyme, and thus detoxify bile salts, has often been included as a criterion for selection of probiotic strain (45). BSH catalyzes the deconjugation of bile salts to liberate free bile acids (46). Strains neither belonging to *P. pentosaceus* nor to *B. coagulans* were positive for BSH activity. On the contrary, strains belonging to *E. mundtii* and *E. faecium* were able to hydrolyze the bile salt TDCA along with *E. hirae* strain 78*.* The BSH activity indicates that the isolates not only survive toxicity of these salts but also de-conjugate these salts and may help in the intestinal colonization of cells and in reducing blood cholesterol level in the host (47).

We tested five selected strains belonging to *P. pentosaceus* for their ability to grow in a fava bean medium with no additional carbon, thus indicating their potential to ferment a bean beverage. Since strains belonging to *B. coagulans* are mainly of value as spores and not as fermenting organisms, and because of the uncertainties regarding *Enterococcus* during fermentation, these strains were excluded from fermentation. The five *P. pentosaceus* strains that were used for fermentation in this study had a similar growth pattern: an initial lag phase followed by exponential growth until 8 h of incubation, when the cells reached a stationary phase.

Finally, we showed the ability of one of the isolated *P. pentosaceus* strains to improve the reduction of phytate by the yeast strain *P. kudriavzevii* TY1322 in a fava bean medium. The origin of the parent strain TY13 and its properties regarding phytase production are described in the previous work by Hellström et al (48, 49, 50). The symbiotic effect of *P. pentosaceus* strain 77 resulted in a 69% larger reduction compared to the monoculture and a 124% higher cell count of *P. kudriavzevii* TY1322 after 48 h of fermentation. This significantly improves the bioavailability of minerals such as iron and zinc, since phytate is a major inhibitor of iron and zinc absorption. This is especially important for individuals following a plant-based diet that does not contain dietary sources of heme iron.

*P. pentosaceus* strains have been previously isolated from legumes such as fava beans (51) and other unspecified beans (52). These findings are in line with our results. Strains of *Pediococcus* are currently used in the food industry as starter cultures for sausages, sauerkraut, cucumber, green bean, and soy sauce fermentation (53). Similarly, specific strains of *Pediococcus* are also used as probiotics for monogastric animals (54). The *Pediococcus* strains isolated in this study were able to use nutrients from fava beans, supporting their potential role as starters for further investigation and their possible use in the non-dairy sector of food industry.

## Conclusion

Some of the strains isolated can be of interest from the perspective of food technology as potential starters for fermentation of non-dairy legume-based products, and others may have potential to be probiotic candidate strains. Further investigation on probiotic properties are needed, such as adherence to gastric mucosa. Five strains showed tolerance to all three conditions tested (survival through GIT, tolerance to bile and phenol, and low pH). Three of these strains belong to *B. coagulans* (strains 51, 221, and 225), and the other two belong to *P. pentosaceus* (strains 77 and 176), which are of interest regarding fermentation, presenting an interesting possibility to produce a combined fermented and probiotic dairy-free food product. The *P. pentosaceus* strains that were isolated in the present study were suitable for the fermentation of a legume beverage. One of the *P. pentosaceus* strains was also able to improve the reduction of phytate by the phytase producing yeast strain *P. kudriavzevii* TY1322 during co-fermentation as well as increase the final cell count of yeast strain. This is a positive indication that new and improved fermented non-dairy products could be produced for commercial use and that strains with probiotic potential can be selected.
